# Editorial for the Special Issue: “Aerogel Hybrids and Nanocomposites”

**DOI:** 10.3390/gels9100812

**Published:** 2023-10-12

**Authors:** István Lázár, Melita Menelaou

**Affiliations:** 1 Department of Inorganic and Analytical Chemistry, University of Debrecen, 4032 Debrecen, Hungary; 2 Department of Chemical Engineering, Cyprus University of Technology, Limassol 3036, Cyprus

Aerogel materials are porous ultralight solid materials obtained from gels, wherein a gas, commonly air, replaces the liquid component. These aerogels exhibit several distinctive characteristics, such as high porosity (exceeding 90% of the total volume), very low apparent density, very high specific surface area and high mechanical strength, when compared to the density of the material, among others. The term “aerogel” encompasses a broad range of structures and does not impose specific restrictions on materials selection or synthetic methodologies. Moreover, aerogels may vary widely in their composition and can be of inorganic or organic origin. Due to their extremely low density, huge specific surface area, and open mesoporous structure, single-component aerogels have already claimed dozens of applications, such as in catalysis, aerospace, construction industries, energy storage devices, solar-steam generation, and medical applications.

Aerogel materials exhibit a wide range of classifications based on their appearance (i.e., monoliths, powders, films), their microstructural characteristics (i.e., microporous, mesoporous, mixed porous), their composition (i.e., inorganic, organic, hybrid materials) and on their polarity and surface functionality (i.e., hydrophilic, hydrophobic, amphiphilic, oleophilic or oleophobic). Typical inorganic aerogels can derive from various oxides like silica and alumina. Organic aerogels comprise carbon-based materials like cellulose, graphene, polymers and chitosan. Hybrid aerogels are prepared by combining organic and inorganic materials at the molecular level, and harnessing the versatile and cooperating properties arising from their constituent components.

Considering these factors, this *Gels* Special Issue focuses on the synthesis, production, structure, properties and applications of such complex aerogel materials, while paying particular attention to the cooperation between the hybrid matrix components and the guest particles. We explored original contributions based on conventional and non-conventional approaches to obtain hybrid and composite aerogel materials. The aim was to delve into fundamental and applied aspects, including the technological and biomedical applications of such materials.

The recent advancements concerning the role of aerogel-based materials in bone and cartilage tissue engineering were thoroughly discussed by Lazar and colleagues. Through their literature review, the authors presented a comprehensive list and summary of various aerogel building blocks and their biological activities synthesized under different synthetic protocols, as well as how the complexity of aerogel scaffolds can influence their in vivo performance, ranging from simple single-component or hybrid aerogels to more intricate and organized structures. Lastly, the authors acknowledged the challenges of aerogel-based hard tissue engineering materials in the near and far future, and their potential connection with emerging healing techniques [1].

In addition, chronic wounds are physical traumas that significantly impair the quality of life of over 40 million patients worldwide, and aerogels can act as carriers for the local delivery of bioactive compounds at the wound site. In this regard, Remuiñán-Pose and colleagues obtained alginate aerogel particles loaded with vancomycin, an antibiotic used for the treatment of Staphylococcus aureus infections, through aerogel technology combined with gel inkjet printing and water-repellent surfaces. Alginate aerogel particles showed high porosity, large surface area, a well-defined spherical shape and a reproducible size. Several drug-loading strategies (in ink, a bath or ink-plus-bath) were tested, which, due to the increase in the specific surface area and the mesoporosity, opened the way to the preparation of more efficient drug-delivery aerogels [2].

Silica and silica-based (hybrid) aerogels represent the most extensively studied family of mesoporous materials. Within this Special Issue, Lee and colleagues published an article on the synthesis of spherical silica aerogel powder with hydrophobic surfaces from a water glass-in-hexane emulsion. The gelation was completed by heating the water glass droplets, which was then followed by the solvent exchange and surface modification steps. The authors claim that the pH of the silicic acid solution was crucial in obtaining a highly porous silica aerogel powder with a spherical morphology [3].

In another study, Pérez-Moreno and colleagues prepared chitosan (CS)-silica xerogel and aerogel hybrids using the sol–gel method, either by direct solvent evaporation at atmospheric pressure or by supercritical drying in CO_2_, respectively. Both types of mesoporous materials exhibited large surface areas (821 m^2^g^−1^–858 m^2^g^−1^), outstanding bioactivity and osteoconductive properties. Through a comparative study, the authors showed that the sol–gel synthesis of CS-silica xerogels and aerogels enhanced not only their bioactive response but also their osteoconduction and cell differentiation properties. Therefore, these new biomaterials can provide adequate secretion of the osteoid for fast bone regeneration [4].

Additionally, Sai et al. described the synthesis of robust fibrous silica-bacterial cellulose (BC) composite aerogels with high performance, following a novel synthetic path. Silica sol was diffused into a fiber-like matrix, which was obtained by cutting the BC hydrogel. A secondary shaping formed a composite wet gel fiber with a nanoscale interpenetrating network structure. The tensile strength of the resulting aerogel fibers reached up to 5.4 MPa. The quantity of BC nanofibers in the unit volume of the matrix was improved significantly by the secondary shaping process. Thus, a novel method was proposed herein to prepare aerogel fibers with excellent performance to meet the requirements of wearable applications [5].

Furthermore, Győri and colleagues reported a way of functionalizing porphyrin rings of 5,10,15,20-tetrakis(4-aminophenyl)porphyrin with a silane linker, followed by complexation with selected metal ions, such as copper and iron, and binding the complexes to the silica aerogel matrix with strong covalent bonds. The as-prepared aerogel catalysts were highly compatible with the aqueous phase, in contrast to the insoluble nature of the porphyrin complexes initially synthesized. The authors studied their catalytic activities in the mineralization reaction of environmental pollutants phenol, 3-chlorophenol and 2,4-dichlorophenol with hydrogen peroxide. The as-obtained catalysts had a large specific surface area and an open mesoporous structure, essential features for heterogeneous catalysis [6].

In another study, Kaplin and colleagues obtained luminescent aerogels in a supercritical carbon dioxide medium for the first time based on sodium alginate, cross-linked with ions of rare earth elements (Eu^3+^, Tb^3+^, Sm^3+^) where phenanthroline, thenoyltrifluoroacetone, dibenzoylmethane and acetylacetonate served as ligands upon SC impregnation. The intensity of the luminescence bands changed after impregnation, while the nature of the influence of the organic additives (ligands) on the luminescent properties of REE ions depended on the nature of both the ion and the ligand. Thus, the authors demonstrated that, upon SC impregnation, ligands could penetrate and act as luminescence sensitizers of rare earth ions throughout the entire thickness of aerogels [7].

The removal of polyvalent metal ions Eu(III) and Th(IV) from aqueous solutions using polyurea-crosslinked calcium alginate (X-alginate) aerogels was investigated, through batch-type experiments under ambient conditions and pH 3, by Georgiou and colleagues. Compared to other materials used for the sorption of Eu(III), such as carbon-based materials, the authors proved for the first time that X-alginate aerogels showed by far the highest sorption capacity. Regarding Th(IV) species, X-alginate aerogels showed the highest capacity per volume among the aerogels reported in the literature. Eu(III) and Th(IV) could be recovered from the beads by 65% and 70%, respectively. Thus, such characteristics, along with their stability in aqueous environments, make X-alginate aerogels attractive candidates for water treatment and metal recovery applications [8].

Carbon materials, including graphene aerogels, carbon nanotube aerogels, and carbon aerogels, have been the subjects of extensive investigation. Kubovics and colleagues fabricated composites involving reduced graphene oxide (rGO) aerogels supporting Pt/TiO_2_ nanoparticles using a one-pot supercritical CO_2_ gelling and drying method, where 3D monolithic aerogels with a mesa/macroporous morphology were obtained, targeted to evaluate the photocatalytic production of H_2_ from methanol in aqueous media. The reaction conditions, aerogel composition and architecture were the factors that varied in optimizing the process. Using methanol as the sacrificial agent, the measured H_2_ production rate for the optimized system was remarkably higher than the values found in the literature for similar Pt/TiO_2_/rGO catalysts and reaction media [9].

Furthermore, Cheng et al. reported a novel method of increasing the mechanical properties of poly(vinyl alcohol) (PVA) aerogels while decreasing their flammabilities, maintaining low densities and using a low-cost/toxicity additive. Sodium hydroxide (NaOH) was used as a base catalyst at flame temperatures to reduce the flammability of PVA aerogels. Low additive levels of NaOH were found to profoundly alter the aerogel properties, such as their mechanical properties and flammability, minimizing their impact on product density without using char-forming agents or halogen compounds to decrease flammability [10].

In summary, aerogel hybrids and nanocomposites represent distinctive materials with versatile applications across various technological and biomedical domains. Among these, silica aerogel stands out as the most prevalent, initially finding commercial use in thermal insulation blankets. However, research endeavors worldwide have since been dedicated to exploring various materials and structures and alternative applications for aerogel-based materials, extending their utility into sectors such as catalysis and tissue engineering. This wide-ranging applicability hinges on the chosen synthesis method, the constituents and the unique properties of the resultant aerogels. It is evident that these materials offer substantial insights, both in terms of fundamental scientific understanding and practical applications, holding the promise of continued discoveries and innovations.
